# ﻿Species diversity and taxonomy of *Vararia* (Russulales, Basidiomycota) with descriptions of six species from Southwestern China

**DOI:** 10.3897/mycokeys.103.118980

**Published:** 2024-03-22

**Authors:** Yinglian Deng, Sana Jabeen, Changlin Zhao

**Affiliations:** 1 The Key Laboratory of Forest Resources Conservation and Utilization in the South-west Mountains of China Ministry of Education, Key Laboratory of National Forestry and Grassland Administration on Biodiversity Conservation in Southwest China, Yunnan Provincial Key Laboratory for Conservation and Utilization of In-forest Re-source, Southwest Forestry University, Kunming 650224, China Southwest Forestry University Kunming China; 2 College of Biodiversity Conservation, Southwest Forestry University, Kunming 650224, China University of Education Lahore Pakistan; 3 Department of Botany, Division of Science and Technology, University of Education, Township, Lahore, Punjab, Pakistan Southwest Forestry University Kunming China; 4 Yunnan Academy of Biodiversity, Southwest Forestry University, Kunming 650224, China University of Education Lahore Pakistan

**Keywords:** Biodiversity, China, phylogenetic analyses, taxonomy, wood-inhabiting fungi, Yunnan Province

## Abstract

*Vararia* is a species-rich genus in the family Peniophoraceae and has been shown to be polyphyletic. In this study, sequences of ITS and LSU rRNA markers of the studied samples were generated and phylogenetic analyses were performed with the maximum likelihood, maximum parsimony, and Bayesian inference methods. Seventeen lineages including six new species from China, i.e., *V.fissurata*, *V.lincangensis*, *V.punctata*, *V.isabellina*, *V.sinensis*, and *V.yaoshanensis* were recognized, in which *V.fissurata* is characterized by the brittle basidiomata with pruinose and cracking hymenophore having white to olivaceous buff hymenial surface, the clamped generative hyphae, presence of the two types gloeocystidia; *V.lincangensis* is characterized by the simple-septa generative hyphae, and thick-walled skeletal hyphae, and ellipsoid basidiospores; *V.punctata* is delimited by its thin to slightly thick-walled generative hyphae, and thick-walled skeletal hyphae, present thick-walled, clavate to cylindrical gloeocystidia; *V.isabellina* is characterized by having the cream to isabelline to slightly brown hymenial surface, thin to slightly thick-walled generative hyphae, and sub-fusiform to navicular basidiospores; *V.sinensis* is distinguishable by its white to slightly pink hymenial surface, thick-walled skeletal hyphae, and sub-fusiform to navicular basidiospores; *V.yaoshanensis* is characterized by cream to pinkish buff to cinnamon-buff hymenial surface, slightly thick-walled generative hyphae, the presence of two types gloeocystidia, and slightly thick-walled, ellipsoid basidiospores. Phylogram based on the ITS+nLSU rDNA gene regions included nine genera within the family Peniophoraceae as *Amylostereum*, *Asterostroma*, *Baltazaria*, *Dichostereum*, *Michenera*, *Peniophora*, *Scytinostroma* and *Vararia*, in which the six new wood-inhabiting fungi species were grouped into genus *Vararia*. The phylogenetic tree inferred from the combined ITS and LSU tree sequences highlighted that *V.fissurata* was found to be the sister to *V.ellipsospora* with strong supports. Additionally, *V.lincangensis* was clustered with *V.fragilis*. Furthermore, *V.punctata* was retrieved as a sister to *V.ambigua*. Moreover, *V.sinensis* was grouped with five taxa as *V.breviphysa*, *V.pirispora*, *V.fusispora*, *V.abortiphysa* and *V.insolita*. The new species *V.isabellina* formed a monophyletic lineage, in which it was then grouped closely with *V.daweishanensis*, and *V.gracilispora*. In addition, *V.yaoshanensis* was found to be the sister to *V.gallica* with strong supports. The present results increased the knowledge of *Vararia* species diversity and taxonomy of corticioid fungi in China. An identification key to 17 species of *Vararia* in China is provided.

## ﻿Introduction

Fungi represent one of the most diverse groups of organisms on the earth, with an indispensable role in the processes and functioning of forest ecosystems (Hyde 2022). The genus *Vararia* P. Karst. belongs to the family Peniophoraceae of the order Russulales (Larsson and Larsson 2003; Miller et al. 2006; Larsson 2007). The Russulales is a well-known order that contains morphologically diverse mushrooms (Miller et al. 2006). Species from this order comprise many representative wood-inhabiting fungal taxa, including hydnoid, corticioid, and polyporoid basidiomes with diverse hymenophoral and cystidial morphology (Yurchenko and Wu 2016; Riebesehl and Langer 2017; Yurchenko et al. 2017; Cui et al. 2019; Riebesehl et al. 2019; Jiang et al. 2021; Wu et al. 2022).

The genus *Vararia* is a corticioid wood-inhabiting fungal genus with a wide distribution, typified by *V.investiens* (Schwein.) P. Karst. It was first described by Karsten as a subgenus of *Xerocarpus* P. Karst. for *Xerocarpusalutarius* (Berk. & M. A. Curtis) P. Karst., which was later found to be a synonym of *Raduluminvestiens* Schwein. Karsten raised Xerocarpussubgen.Vararia to the generic rank (Karasinski 2010). The genus is characterized by the resupinate basidiomata, a dimitic hyphal structure with simple-septate or clamped generative hyphae and often dextrinoid dichohyphae in Melzer’s reagent, the presence of gloeocystidia, and variously shaped smooth basidiospores with or without an amyloid reaction (Karnste 1898; Boidin and Lanquetin 1975; Boidin 1980; Bernicchia and Gorjón 2010). The species of *Vararia* are found on fallen angiosperm branches, dead woody or herbaceous stems or occasionally on gymnosperm wood (Yurchenko et al. 2017). Based on the MycoBank database (http://www.mycobank.org, accessed on 17 January 2024) and the Index Fungorum (http://www.indexfungorum.org, accessed on 17 January 2024), *Vararia* has registered 99 speciﬁc and infraspeciﬁc names, and the actual number of the species has reached up to 76, currently known, and they occur mainly in the tropical and subtropical areas of the world (Cunningham 1955; Gilbertson 1965; Boidin 1967; Pouzar 1982; Boidin and Lanquetin 1987; Stalpers 1996; Boidin and Gilles 1999; Larsson and Larsson 2003; Bernicchia and Gorjón 2010; Duhem and Buyck 2012; Sanyal et al. 2012; Nakasone 2015; Liu and He 2016; Dai et al. 2021; Zou et al. 2022; Deng and Zhao 2023).

Classification of the kingdom of fungi has been updated continuously, based on the frequent inclusion of data from DNA sequences in many phylogenetic studies (Yurchenko et al. 2020). These pioneering research studies into the family Peniophoraceae were just the prelude to the molecular systematics period (Zou et al. 2022). The phylogenetic diversity displayed by corticioid fungal species, based on ITS1-5.8S-ITS2-nrLSU nuclear rDNA, revealed that the taxa of Peniophoraceae were nested in the russuloid clade, which holds a considerable share of the phylogenetic framework, and included the genera of *Asterostroma* Massee, *Baltazaria* Leal-Dutra, Dentinger & G.W. Griff., *Dichostereum* Pilát, *Gloiothele* Bres., *Lachnocladium* Lév., *Michenera* Berk. & M.A. Curtis, *Peniophora* Cooke, *Scytinostroma* Donk, *Vesiculomyces* E. Hagstr. and *Vararia* (Larsson and Larsson 2003; Larsson and Larsson 2004; Larsson 2007; Leal-Dutra et al. 2018; Zou et al. 2022; Li et al. 2023). Morphologically, *Scytinostroma* was similar to *Vararia*, which usually differed in having the typical dichohyphae (Bernicchia and Gorjón 2010). The taxonomic distinction between *Scytinostroma* and *Vararia* has been questioned (Hallenberg 1985; Boidin and Lanquetin 1987; Stalpers 1996; Boidin et al 1998). However, there has been general agreement that the two genera were closely related and that they together made up a natural group. Larsson and Larsson (2003) strongly suggested that neither skeletal hyphae nor their branching patterns have any predictive power in a phylogenetic context.

During investigations on the wood-inhabiting fungi in the Yunnan province of China, the samples representing six additional species belonging to genera *Vararia* were collected. To clarify the placement and relationships of the six species, we carried out a phylogenetic and taxonomic study on *Vararia*, based on the ITS and LSU sequences.

## ﻿Materials and methods

### ﻿Morphology

Fresh fruiting bodies of the fungi were collected from Dali, Dehong, Lincang, Puer, Yuxi and Zhaotong of Yunnan Province, P.R. China. Specimens were dried in an electric food dehydrator at 40 °C, then sealed and stored in an envelope bag and deposited in the herbarium of the Southwest Forestry University (SWFC), Kunming, Yunnan Province, P.R. China. Macromorphological descriptions are based on field notes and photos captured in the field and lab. Color terminology follows Petersen (Petersen 1996). Micromorphological data were obtained from the dried specimens when observed under a light microscope following the previous study (Zhao et al. 2023; Guan et al. 2023). The following abbreviations are used: KOH = 5% potassium hydroxide water solution, CB = Cotton Blue, CB– = acyanophilous, IKI = Melzer’s Reagent, IKI– = both inamyloid and indextrinoid, L = mean spore length (arithmetic average for all spores), W = mean spore width (arithmetic average for all spores), Q = variation in the L/W ratios between the specimens studied and n = a/b (number of spores (a) measured from given number (b) of specimens).

### ﻿Molecular phylogeny

The EZNA HP Fungal DNA Kit (Omega Biotechnologies Co., Ltd., Kunming, China) was used to extract DNA with some modifications from the dried specimens. The nuclear ribosomal ITS region was amplified with primers ITS5 and ITS4 (White et al. 1990). The PCR procedure for ITS was as follows: initial denaturation at 95 °C for 3 min, followed by 35 cycles at 94 °C for 40 s, 58 °C for 45 s and 72 °C for 1 min, and a final extension of 72 °C for 10 min. The nuclear LSU region was amplified with primer pair LR0R and LR7 (Vilgalys and Hester 1990; Rehner and Samuels 1994). The PCR procedure for LSU was as follows: initial denaturation at 94 °C for 1 min, followed by 35 cycles at 94 °C for 30 s, 48 °C for 1 min and 72 °C for 1.5 min, and a final extension of 72 °C for 10 min. The PCR procedure for ITS and LSU followed a previous study (Zhao and Wu 2017). All of the newly generated sequences were deposited in NCBI GenBank (https://www.ncbi.nlm.nih.gov/genbank/) (Table 1).

**Table 1.** List of species, specimens and GenBank accession numbers of sequences used in this study. [* Indicates type materials].

Species name	Specimen No.	GenBank accession No.		Country	References
		ITS	nLSU		
*Amylostereumchailletii*	NH8031	AF506406	AF506406	Sweden	Larsson and Larsson 2003
*A.laevigatum*	NH12863	AF506407	AF506407	Sweden	Larsson and Larsson 2003
*Asterostromabambusicola*	He4132	KY263865	KY263871	Thailand	Liu et al. 2017
*A.cervicolor*	He2314	KY263859	KY263869	China	Unpublished
*A.cervicolor*	He4020	KY263860	KY263868	Thailand	Unpublished
*A.muscicola*	He4397	MK625630	MK625563	China	Unpublished
*Baltazariagalactina*	He4999	MK625618	MK625547	China	Unpublished
*B.octopodites*	FLOR63715	MH260042	MH260060	United Kingdom	Leal-Dutra et al. 2018
*Confertobasidiumolivaceoalbum*	FP90196	AF511648	AF511648	Sweden	Larsson and Larsson 2003
*Dichostereumboidinii*	He4410	MH538315	MH538331	China	Vu et al. 2019
*D.boidinii*	He5026	MH538324	MH538330	China	Liu et al. 2019
*D.pallescens*	CBS:718.81	MH861456	MH873198	USA	Vu et al. 2019
*Metulodontianivea*	NH13108	AF506423	AF506423	Sweden	Larsson and Larsson 2003
*Micheneraartocreas*	GHL-2016-Oct	MH204688	MH204691	USA	Liu et al. 2019
*M.incrustata*	He5368	MH204689	MH204690	China	Liu et al. 2019
*Peniophoracinerea*	CBS:261.37	MH855905	MH867412	Belgium	Vu et al. 2019
*P.cinerea*	He3725	MK588769	MK588809	China	Unpublished
*P.incarnata*	CBS 430.72	MH860518	MH872230	Netherlands	Vu et al. 2019
*P.incarnata*	NH10271	AF506425	AF506425	Sweden	Larsson and Larsson 2003
*P.nuda*	LZ15-07	MT859929	—	China	Unpublished
*P.quercina*	CBS 407.50	MH856687	MH868204	France	Vu et al. 2019
*P.quercina*	CBS:410.50	MH856690	MH868207	France	Vu et al. 2019
*Scytinostromaacystidiatum*	He5646	MK625568	MK625494	China	Unpublished
*S.alutum*	CBS:762.81	MH861482	MH873221	France	Vu et al. 2019
*S.beijingensis*	He7768	OQ731943	OQ729731	China	Li et al. 2023
*S.boidinii*	He6911	OQ731934	OQ729724	China	Li et al. 2023
*S.duriusculum*	He3590	MK625571	MK625499	China	Unpublished
*S.hemidichophyticum*	CBS:702.84	MH861818	MH873509	Belgium	Vu et al. 2019
*S.renisporum*	CBS:771.86	MH862051	MH873738	Bali	Vu et al. 2019
*S.subrenisporum*	He4792	MK625566	MK625493	China	Unpublished
*Varariaabortiphysa*	CBS:632.81	MH861387	—	Gabon	Vu et al. 2019
*V.ambigua*	CBS 634.81	MH861388	MH873137	France	Vu et al. 2019
*V.amphithallica*	CBS:635.81	MH861389	MH873138	Gabon	Vu et al. 2019
*V.amphithallica*	CBS:687.81	MH861431	—	France	Vu et al. 2019
*V.aurantiaca*	CBS:641.81	MH861393	—	France	Vu et al. 2019
*V.aurantiaca*	CBS:642.81	MH861394	—	Gabon	Vu et al. 2019
*V.breviphysa*	CBS:643.81	MH873144	MH873144	Gabon	Vu et al. 2019
*V.breviphysa*	CBS:644.81	MH861396	—	Gabon	Vu et al. 2019
*V.calami*	CBS:646.81	MH861398	—	France	Vu et al. 2019
*V.calami*	CBS:648.81	MH861399	—	France	Vu et al. 2019
*V.callichroa*	CBS:744.91	MH874000	MH874000	France	Vu et al. 2019
*V.cinnamomea*	CBS:641.84	MH861794	—	Madagascar	Vu et al. 2019
*V.cinnamomea*	CBS:642.84	MH873488	MH873488	Madagascar	Vu et al. 2019
*V.cremea*	CBS:651.81	MH873147	MH873147	France	Vu et al. 2019
*V.daweishanensis*	CLZhao 17911	OP380613	—	China	Zou et al. 2022
*V.daweishanensis*	CLZhao 17936	OP380614	—	China	Zou et al. 2022
*V.dussii*	CBS:652.81	MH873148	MH873148	France	Vu et al. 2019
*V.dussii*	CBS:655.81	MH861405	—	France	Vu et al. 2019
*V.ellipsospora*	HHB-19503	MW740328	—	New Zealand	Zou et al. 2022
***V.fissurata***	**CLZhao 10118**	**PP083288**	—	**China**	**Present study**
***V.fissurata***	**CLZhao 10181**	**PP083289**	—	**China**	**Present study**
***V.fissurata***	**CLZhao 22538**	**PP083290**	—	**China**	**Present study**
***V.fissurata***	**CLZhao 4614**	**PP083283**	—	**China**	**Present study**
***V.fissurata***	**CLZhao 5218**	**OQ025218**	**OR539502**	**China**	**Present study**
***V.fissurata***	**CLZhao 6070**	**PP083284**	—	**China**	**Present study**
***V.fissurata***	CLZhao 8171*	**OQ025219**	**OR539503**	**China**	**Present study**
***V.fissurata***	**CLZhao 9618**	**PP083285**	—	**China**	**Present study**
***V.fissurata***	**CLZhao 9668**	**PP083286**	—	**China**	**Present study**
***V.fissurata***	**CLZhao 9697**	**PP083287**	—	**China**	**Present study**
*V.fragilis*	CLZhao 16475	OP380612	—	China	Zou et al. 2022
*V.fragilis*	CLZhao 2628	OP380611	—	China	Zou et al. 2022
*V.fusispora*	PDD:119539	OL709443	—	New Zealand	Zou et al. 2022
*V.gallica*	CBS 234.91	MH862250	MH873932	Canada	Vu et al. 2019
*V.gallica*	CBS 656.81	MH861406	MH873152	France	Vu et al. 2019
*V.gillesii*	CBS:660.81	MH873153	MH873153	Cote d’Ivoire	Vu et al. 2019
*V.gomezii*	CBS:661.81	MH873154	MH873154	France	Vu et al. 2019
*V.gracilispora*	CBS:663.81	MH861411	—	Gabon	Vu et al. 2019
*V.gracilispora*	CBS:664.81	MH861412	—	Gabon	Vu et al. 2019
*V.insolita*	CBS:668.81	MH861413	—	France	Vu et al. 2019
*V.intricata*	CBS:673.81	MH861418	—	France	Vu et al. 2019
*V.investiens*	FP-151122ITS	MH971976	—	USA	Liu et al. 2019
*V.investiens*	UC2023140	KP814286	—	USA	Rosenthal et al. 2017
***V.isabellina***	**CLZhao 22852**	**OR048789**	**OR506350**	**China**	**Present study**
***V.isabellina***	**CLZhao 22887**	**OR048788**	**OR506351**	**China**	**Present study**
***V.lincangensis***	CLZhao 22791*	**OR048819**	**OR506348**	**China**	**Present study**
***V.lincangensis***	**CLZhao 22799**	**OR048818**	**OR506349**	**China**	**Present study**
*V.malaysiana*	CBS:644.84	MH873490	MH873490	Singapore	Vu et al. 2019
*V.minispora*	CBS:682.81	MH861426	—	France	Vu et al. 2019
*V.ochroleuca*	CBS:465.61	MH858109	—	France	Vu et al. 2019
*V.ochroleuca*	JS24400	AF506485	AF506485	Norway	Larsson and Larsson 2003
*V.parmastoi*	CBS:879.84	MH861852	MH861852	Uzbekistan	Vu et al. 2019
*V.pectinata*	CBS:685.81	MH861429	—	Cote d’Ivoire	Vu et al. 2019
*V.perplexa*	CBS:695.81	MH861438	—	France	Vu et al. 2019
*V.pirispora*	CBS:720.86	MH862016	—	France	Vu et al. 2019
***V.punctata***	**CLZhao 22423**	**OR048813**	**OR539685**	**China**	**Present study**
***V.punctata***	CLZhao 22439*	**OR048812**	**OR510675**	**China**	**Present study**
*V.rhombospora*	CBS:743.81	MH861470	—	France	Vu et al. 2019
*V.rosulenta*	CBS:743.86	MH862028	—	France	Vu et al. 2019
*V.rugosispora*	CBS:697.81	MH861440	—	Gabon	Vu et al. 2019
*V.sigmatospora*	CBS:748.91	MH874001	MH874001	Netherlands	Vu et al. 2019
***V.sinensis***	CLZhao 25160*	**OR102494**	**OR510678**	**China**	**Present study**
***V.sinensis***	**CLZhao 25161**	**OR102495**	**OR510679**	**China**	**Present study**
*V.sphaericospora*	CBS:700.81	MH873185	MH873185	Gabon	Vu et al. 2019
*V.sphaericospora*	CBS:703.81	MH861446	—	Gabon	Vu et al. 2019
*V.sphaericospora*	He4847	MK625592	MK625521	China	Unpublished
*V.trinidadensis*	CBS:650.84	MH873495	MH873495	Madagascar	Vu et al. 2019
*V.trinidadensis*	CBS:651.84	MH861803	—	Madagascar	Vu et al. 2019
*V.tropica*	CBS 704.81	MH861447	MH873189	France	Vu et al. 2019
*V.vassilievae*	UC2022892	KP814203	—	USA	Unpublished
*V.verrucosa*	CBS:706.81	MH861449	MH861449	France	Vu et al. 2019
***V.yaoshanensis***	**CLZhao 20528**	**PP091673**	—	**China**	**Present study**
***V.yaoshanensis***	**CLZhao 20531**	**PP091674**	—	**China**	**Present study**
***V.yaoshanensis***	**CLZhao 20565**	**PP091675**	**PP091683**	**China**	**Present study**
***V.yaoshanensis***	**CLZhao 20605**	**PP091676**	—	**China**	**Present study**
***V.yaoshanensis***	**CLZhao 20608**	**PP091677**	—	**China**	**Present study**
***V.yaoshanensis***	**CLZhao 20617**	**PP091678**	—	**China**	**Present study**
***V.yaoshanensis***	**CLZhao 20619**	**PP091679**	—	**China**	**Present study**
***V.yaoshanensis***	**CLZhao 20624**	**PP091680**	—	**China**	**Present study**
***V.yaoshanensis***	**CLZhao 20646**	**PP091681**	—	**China**	**Present study**
***V.yaoshanensis***	**CLZhao 20656**	**PP091682**	—	**China**	**Present study**
***V.yaoshanensis***	**CLZhao 20669**	**PP091666**	—	**China**	**Present study**
***V.yaoshanensis***	**CLZhao 20677**	**PP091667**	—	**China**	**Present study**
***V.yaoshanensis***	CLZhao 20693*	**PP091665**	**PP091684**	**China**	**Present study**
***V.yaoshanensis***	**CLZhao 20697**	**PP091668**	—	**China**	**Present study**
***V.yaoshanensis***	**CLZhao 20709**	**PP091669**	—	**China**	**Present study**
***V.yaoshanensis***	**CLZhao 20713**	**PP091670**	—	**China**	**Present study**
***V.yaoshanensis***	**CLZhao 20717**	**PP091671**	—	**China**	**Present study**
***V.yaoshanensis***	**CLZhao 20724**	**PP091672**	—	**China**	**Present study**

**Figure 1.**
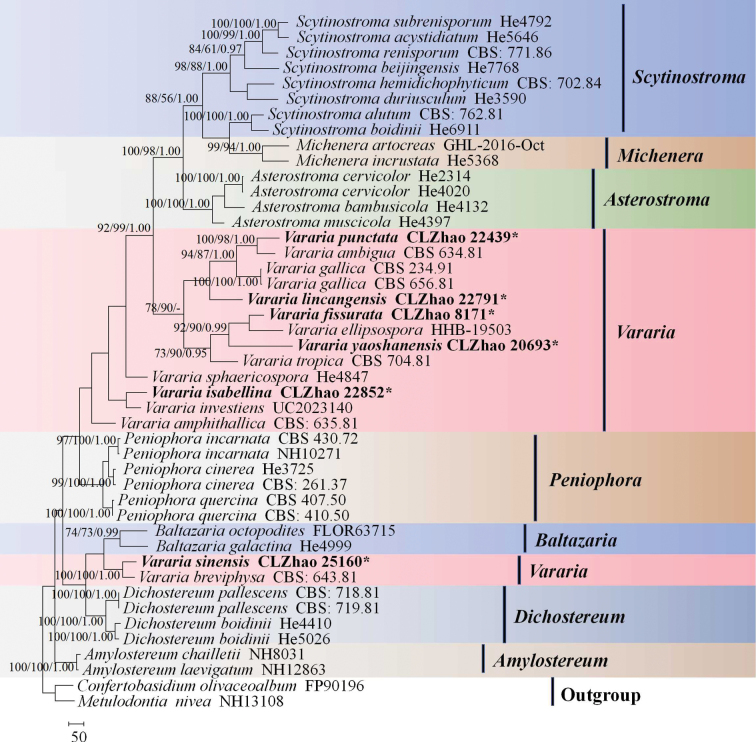
Maximum parsimony strict consensus tree illustrating the phylogeny of *Vararia* and related genera in the family Peniophoraceae based on ITS+LSU sequences. Branches are labelled with maximum likelihood bootstrap values > 70%, parsimony bootstrap values > 50% and Bayesian posterior probabilities > 0.95, respectively.

**Figure 2.**
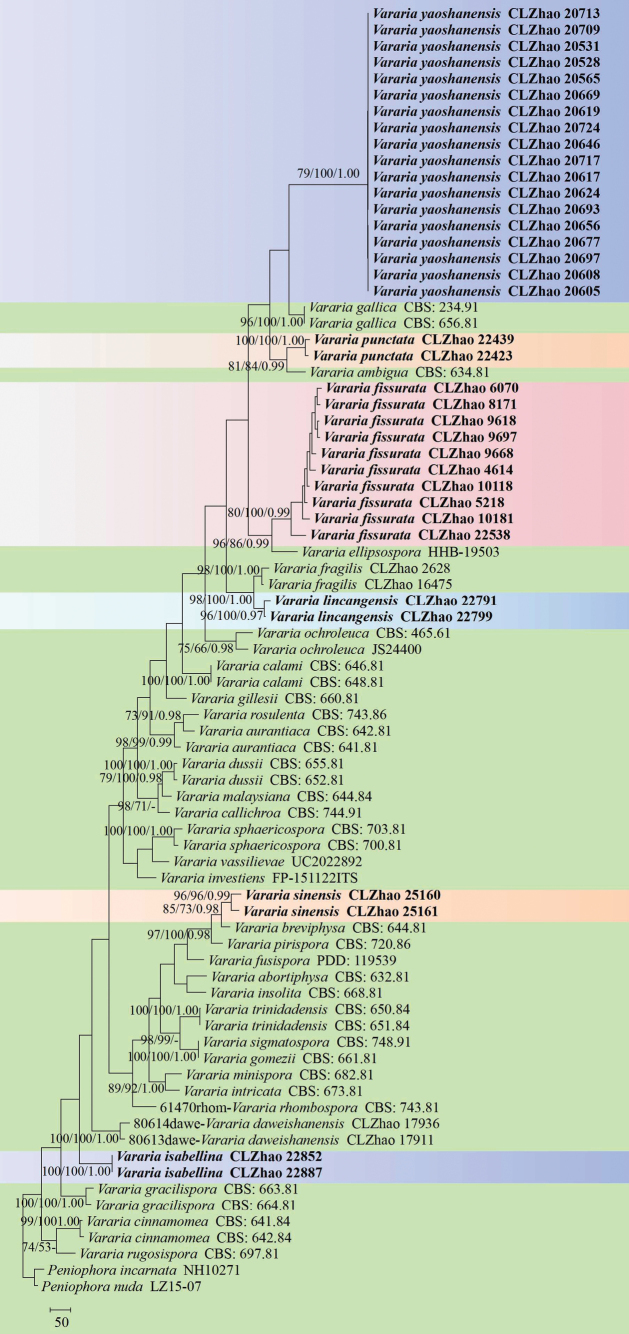
Maximum parsimony strict consensus tree illustrating the phylogeny of the two new species and related species in *Vararia*, based on ITS sequences. Branches are labelled with maximum likelihood bootstrap values > 70%, parsimony bootstrap values > 50% and Bayesian posterior probabilities > 0.95, respectively.

The sequences were aligned in MAFFT version 7 (Katoh et al. 2019) using the G-INS-i strategy. The alignment was adjusted manually using AliView version 1.27 (Larsson 2014). Sequences of *Confertobasidiumolivaceoalbum* (Bourdot & Galzin) (AF511648) Jülich and *Metulodontianivea* (P. Karst.) Parmasto () retrieved from GenBank were used as the outgroups in the ITS+LSU analysis (Fig. 1); Sequences of *Peniophoraincarnata* (Pers.) P. Karst. (AF506425) and *Peniophoranuda* (Fr.) Bres. (MT859929) retrieved from GenBank were used as the outgroups in the ITS analysis (Fig. 2) (Leal-Dutra et al. 2018; Zhao et al. 2021).

Maximum parsimony (MP), Maximum Likelihood (ML), and Bayesian Inference (BI) analyses were applied to the combined three datasets following a previous study (Zhao and Wu 2017). All characters were equally weighted and gaps were treated as missing data. Trees were inferred using the heuristic search option with TBR branch swapping and 1,000 random sequence additions. Max-trees were set to 5,000, branches of zero length were collapsed and all parsimonious trees were saved. Clade robustness was assessed using bootstrap (BT) analysis with 1,000 pseudo replicates (Felsenstein 1985). Descriptive tree statistics – tree length (TL), composite consistency index (CI), composite retention index (RI), composite rescaled consistency index (RC) and composite homoplasy index (HI) – were calculated for each maximum parsimonious tree generated. The combined dataset was also analysed using Maximum Likelihood (ML) in RAxML-HPC2 through the CIPRES Science Gateway (Miller et al. 2012). Branch support (BS) for the ML analysis was determined by 1000 bootstrap pseudo replicates.

MrModeltest 2.3 (Nylander 2004) was used to determine the best-ﬁt evolution model for each dataset for the purposes of Bayesian inference (BI) which was performed using MrBayes 3.2.7a with a GTR+I+G model of DNA substitution and a gamma distribution rate variation across sites (Ronquist et al. 2012). A total of four Markov chains were run for two runs from random starting trees for 1.2 million generations for ITS+LSU (Fig. 1); and 4 million generations for ITS (Fig. 2) with trees and parameters sampled every 1,000 generations. The ﬁrst quarter of all the generations were discarded as burn-ins. A majority rule consensus tree was computed from the remaining trees. Branches were considered as significantly supported if they received a maximum likelihood bootstrap support value (BS) of > 70%, a maximum parsimony bootstrap support value (BT) of > 70% or a Bayesian posterior probability (BPP) of > 0.95.

## ﻿Results

### ﻿Molecular phylogeny

The ITS+LSU dataset (Fig. 1) comprised sequences from 45 fungal specimens representing 38 taxa. The dataset had an aligned length of 2,304 characters, of which 1,181 characters were constant, 346 were variable and parsimony-uninformative and 777 (50%) were parsimony-informative. Maximum parsimony analysis yielded 3 equally parsimonious trees (TL = 5,051, CI = 0.3985, HI = 0.6015, RI = 0.5522 and RC = 0.2201). The best model of nucleotide evolution for the ITS+LSU dataset estimated and applied in the Bayesian analysis was found to be GTR+I+G. Bayesian analysis and ML analysis resulted in a similar topology as in the MP analysis. The Bayesian analysis had an average standard deviation of split frequencies = 0.004451 (BI) and the effective sample size (ESS) across the two runs is double the average ESS (avg. ESS) = 324. The phylogram based on the ITS+LSU rDNA gene regions (Fig. 1) included eight genera within Peniophoraceae (Russulales), which were *Asterostroma*, *Amylostereum*, *Baltazaria*, *Dichostereum*, *Michenera*, *Peniophora*, *Scytinostroma* and *Vararia*, in which six new species were grouped into the genera *Vararia*.

The ITS dataset (Fig. 2) comprised sequences from 79 fungal specimens representing 38 taxa. The dataset had an aligned length of 849 characters, of which 199 characters were constant, 65 were variable and parsimony-uninformative and 585 (50%) were parsimony-informative. Maximum parsimony analysis yielded 1 equally parsimonious tree (TL = 4,058, CI = 0.3233, HI = 0.6767, RI = 0.7299 and RC = 0.2360). The best model of nucleotide evolution for the ITS dataset estimated and applied in the Bayesian analysis was found to be GTR+I+G. Bayesian analysis and ML analysis resulted in a similar topology as in the MP analysis. The Bayesian analysis had an average standard deviation of split frequencies = 0.001947 (BI) and the effective sample size (ESS) across the two runs is double the average ESS (avg. ESS) = 888. The phylogenetic tree (Fig. 2), inferred from the ITS sequences, highlighted that *V.fissurata* was the sister to *V.ellipsospora* G. Cunn. with strong supports. The new species *V.lincangensis* was clustered with *V.fragilis* L. Zou & C.L. Zhao. Furthermore, *V.punctata* was retrieved as a sister to *V.ambigua* Boidin, Lanq. & Gilles. Moreover, *V.isabellina* formed a monophyletic lineage, and it was then grouped closely with *V.daweishanensis* L. Zou & C.L. Zhao, and *V.gracilispora* Boidin & Lanq. The species *V.sinensis* was grouped with five taxa as *Varariabreviphysa* Boidin & Lanq., *V.pirispora* Boidin, Gilles & Lanq., *V.fusispora* G. Cunn., *V.abortiphysa* Boidin & Lanq., and *V.insolita* Boidin & Lanq. In addition, *V.yaoshanensis* was sister to *V.gallica* (Bourdot & Galzin) Boidin with strong supports.

### ﻿Taxonomy

#### ﻿ Vararia fissurata

Taxon classificationFungiRussulalesLachnocladiaceae

Y.L. Deng & C.L. Zhao sp. nov.

FCDF3E2C-FA46-5797-9156-FBE15AE7DBA9

MB851793

Figs 3 , 4

##### Holotype.

China. Yunnan Province, Yuxi, Xinping County, the Ancient Tea Horse Road, 23°57'10"N, 101°30'41"E, altitude 2600 m a.s.l., on the trunk of angiosperm, leg. C.L. Zhao, 21 August 2018, CLZhao 8171 (SWFC).

**Figure 3.**
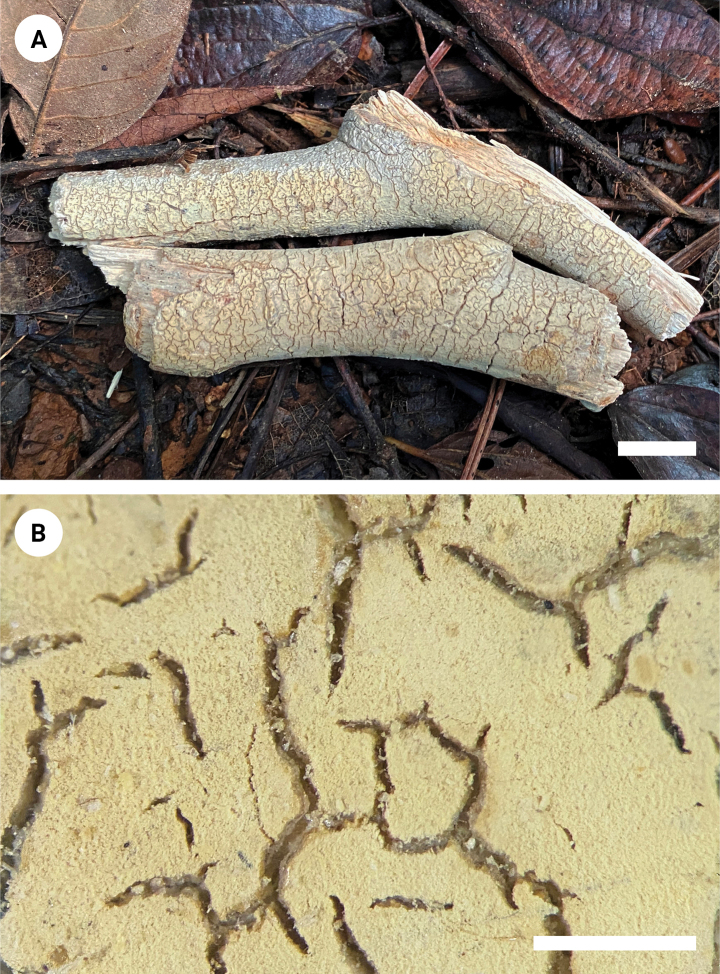
Basidiomata of *Varariafissurata* (holotype). Scale bars: 1 cm (**A**); 1 mm (**B**).

##### Etymology.

*Fissurata* (Lat.): referring to the cracking hymenial surface.

**Figure 4.**
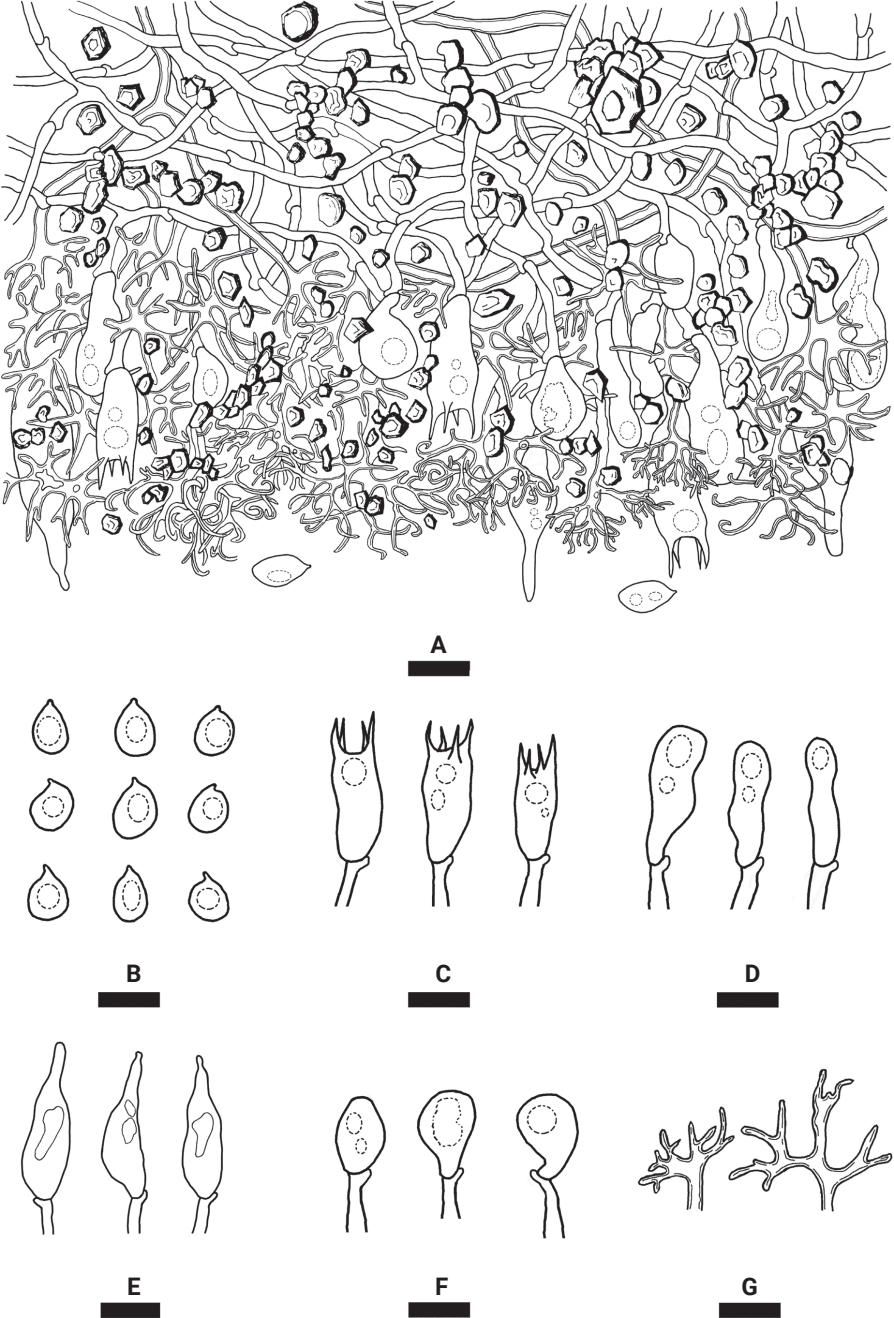
Microscopic structures of *Varariafissurata* (holotype) **A** a section of hymenium **B** basidiospores **C** basidia **D** basidioles **E** gloeocystidia subulate **F** gloeocystidia subglobose **G** dichohyphae. Scale bars: 10 µm (**A–G**).

##### Description.

Basidiomata annual, resupinate, adnate, pruinose, brittle, without odor or taste when fresh, up to 12 cm long, 2.5 cm wide, and 100 µm thick. Hymenial surface smooth, white to olivaceous buff when fresh, and olivaceous buff upon drying, sparsely and deeply cracked with age. Sterile margin distinct, white, and up to 2 mm wide.

Hyphal system dimitic, generative hyphae with clamp connections, colorless, thin-walled, moderately branched, interwoven, 2–3 µm in diameter; IKI–, CB–, tissues unchanged in KOH. Dichohyphae predominate, yellowish, capillary, frequently branched, 1.5 µm in diameter, thick-walled, dichotomously to irregularly branched with main branches and acute tips, weakly to moderately dextrinoid in Melzer’s reagent, CB–, tissues unchanged in KOH; subhymenial hyphae densely covered by a lot of bulk crystals.

Gloeocystidia empty or filled with refractive flocculent matter, two types: (1) Gloeocystidia subglobose, colorless, thin-walled, smooth, 11–23 × 6–12 µm; (2) Gloeocystidia subulate, usually containing refractive materials; slightly constricted at the neck, colorless, thin-walled, smooth, 25.5–43 × 7–11 µm. Basidia cylindrical, with four sterigmata and a basal clamp connection, 20–27 × 4–8 µm; basidioles dominant, in shape similar to basidia but slightly smaller.

Basidiospores ellipsoid to broadly ellipsoid, colorless, thin-walled, smooth, IKI–, CB–, 5–10 × 3–7 µm, L = 7.37 µm, W = 5.22 µm, Q = 1.38–1.44 (n = 150/5).

##### Additional specimens examined

**(paratypes).** China. Yunnan Province, Yuxi, Xinping County, the Ancient Tea Horse Road, 23°57'10"N, 101°30'41"E, altitude 2600 m a.s.l., on fallen angiosperm branch, leg. C.L. Zhao, 13 January 2018, CLZhao 5218 (SWFC); Puer, Zhenyuan County, Heping Town, Damoshan, 23°56'21"N, 101°25'32"E, altitude 2240 m a.s.l., on fallen angiosperm branch, leg. C.L. Zhao, 16 January 2018, CLZhao 6070 (SWFC); Dali, Weishan Country, Qinghua Town, Green Peacock Nature Reserve, 25°23'35"N, 100°31'39"E, altitude 1500 m a.s.l., on the fallen branch of angiosperm, leg. C.L. Zhao, 18 July 2022, CLZhao 22538 (SWFC); Puer, Jingdong County, Wuliangshan National Nature Reserve, 24°34'45"N, 100°830'03"E, altitude 2000 m a.s.l., on fallen angiosperm branch, leg. C.L. Zhao, 6 October 2017, CLZhao 4614 (SWFC); 6 January 2019, CLZhao 9618, CLZhao 9668 and CLZhao 9697 (SWFC); Dali, Nanjian County, Lingbaoshan National Forest Park, 24°78'26"N, 100°51'30"E, altitude 2500 m a.s.l., on fallen angiosperm branch, leg. C.L. Zhao, 9 January 2019, CLZhao 10118, and CLZhao 10181 (SWFC).

#### ﻿ Vararia isabellina

Taxon classificationFungiRussulalesLachnocladiaceae

Y.L. Deng & C.L. Zhao sp. nov.

629640AE-1559-513F-80F5-12C7DF651083

MB851798

Figs 5 , 6

##### Holotype.

China. Yunnan Province, Lincang, Fengqing County, 24°67'18"N, 100°19'67"E, altitude 1660 m a.s.l., on the fallen angiosperm branch, leg. C.L. Zhao, 20 July 2022, CLZhao 22852 (SWFC).

**Figure 5.**
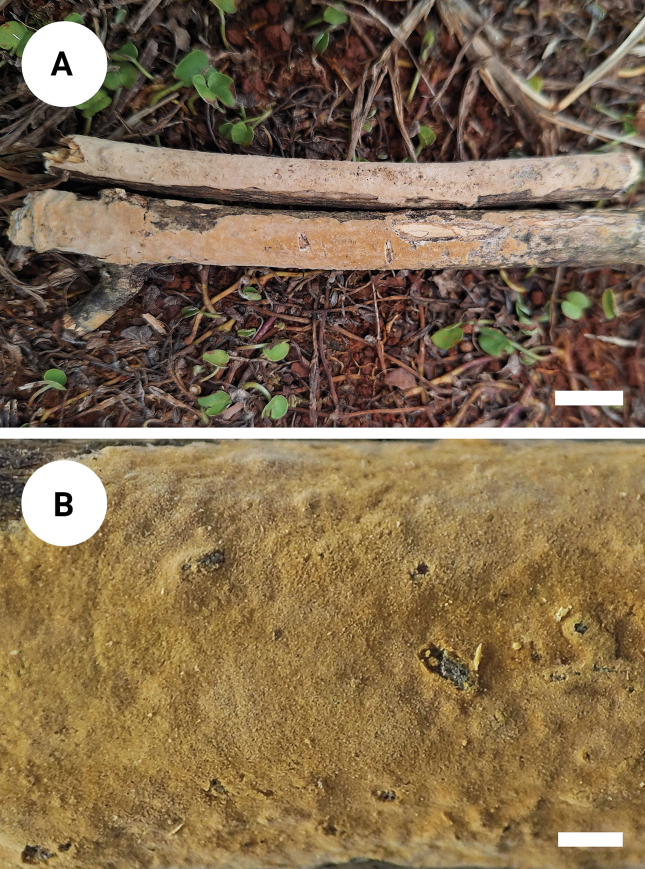
Basidiomata of *Varariaisabellina* (holotype). Scale bars: 1 cm (**A**); 1 mm (**B**).

##### Etymology.

*Isabellina* (Lat.): referring to the isabelline to yellowish-brown basidiomata.

**Figure 6.**
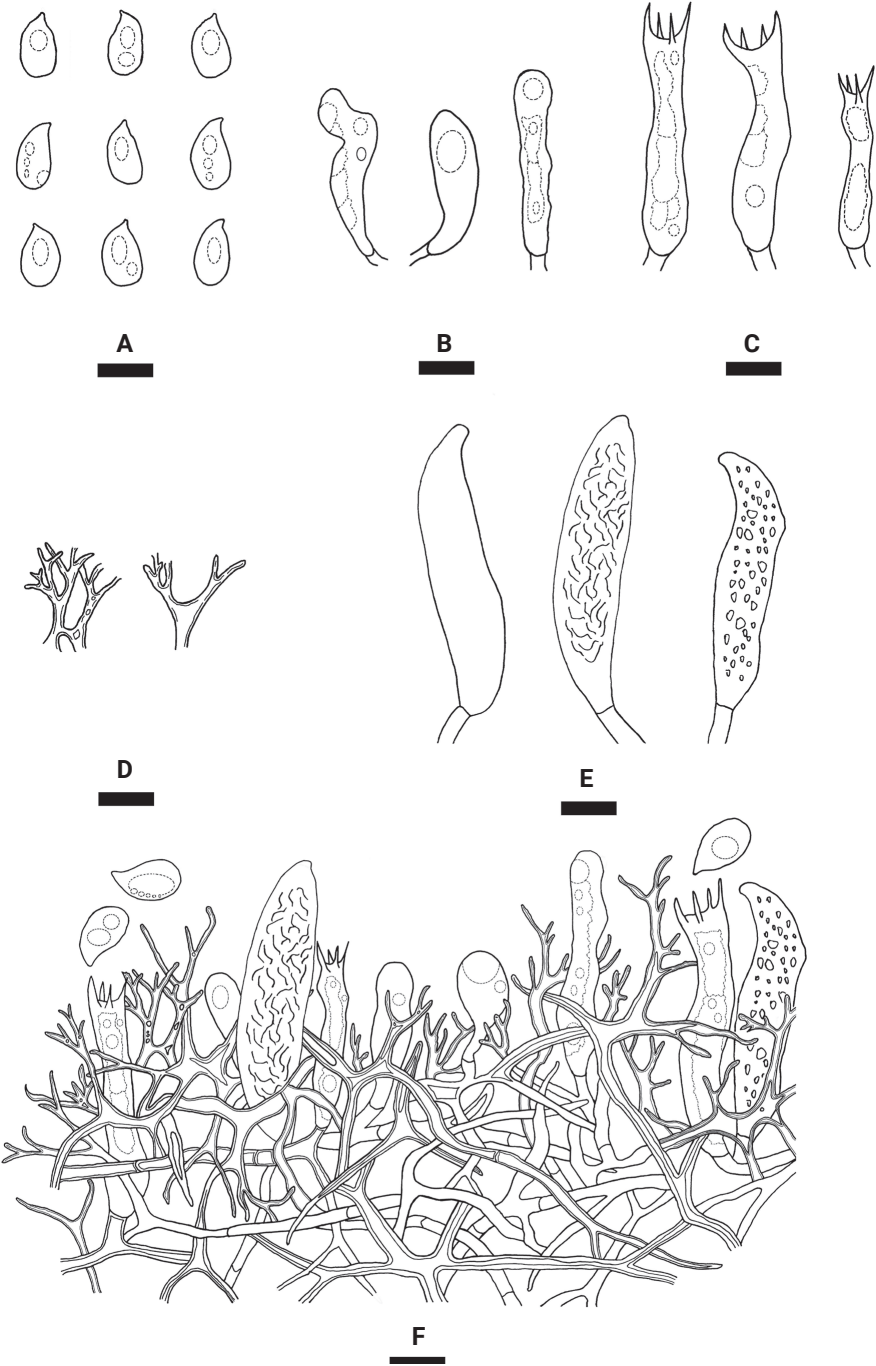
Microscopic structures of *Varariaisabellina* (holotype) **A** basidiospores **B** basidioles **C** basidia **D** dichohyphae **E** gloeocystidia **F** a section of hymenium. Scale bars: 10 µm (**A–F**).

##### Description.

Basidiomata annual, membranous, soft, and adnate, without odor or taste when fresh, up to 90 mm long, 10 mm wide, and 50–90 µm thick. Hymenial surface smooth, cream to isabelline when fresh, isabelline to slightly brown when dry. Sterile margin thinning out, cream to isabelline, and up to 1 mm wide.

Hyphal system dimitic, generative hyphae bearing simple-septa, colorless, thin to slightly thick-walled, frequently branched, 2.5–4 µm in diameter, IKI–, CB–, tissues unchanged in KOH. Dichohyphae predominant, yellowish, distinctly thick-walled, dichotomously to irregularly branched with main branches up to 4 μm in diameter and with acute tips, moderately dextrinoid in Melzer’s reagent, CB–, tissues unchanged in KOH; dichohyphae in hymenium similar to those in subiculum but more branched, with more narrow and shorter branches, with slightly curved tips and stronger.

Gloeocystidia spindle to subcylindrical, smooth, colorless, thin-walled, usually containing refractive materials, 38–47 × 8–13 μm. Basidia subcylindrical, slightly constricted at the neck, with four sterigmata and a basal simple septum connection, 33–39 × 7–9 μm; basidioles dominant, in shape similar to basidia, but slightly smaller.

Basidiospores sub-fusiform to navicular, colorless, smooth, with numerous oil-drops, thin-walled, IKI–, CB–, 9–13 × 5–8 µm, L = 11.66 µm, W = 6.69 µm, Q = 1.68–1.78 (n = 60/2).

##### Additional specimen examined

**(paratype).** China. Yunnan Province, Lincang, Fengqing County, 24°67'18"N, 100°19'67"E, altitude 1660 m a.s.l., on the fallen angiosperm branch, leg. C.L. Zhao, 20 July 2022, CLZhao 22887 (SWFC).

#### ﻿ Vararia lincangensis

Taxon classificationFungiRussulalesLachnocladiaceae

Y.L. Deng & C.L. Zhao sp. nov.

09ECE14E-80AA-5E44-A18A-DEDEBA45397A

MB851794

Figs 7 , 8

##### Holotype.

China. Yunnan Province, Lincang, Fengqing County, Yaojie Township, Xingyuan Village, 24°61'44"N, 100°17'21"E, altitude 1660 m a.s.l., on the fallen angiosperm branch, leg. C.L. Zhao, 20 July 2022, CLZhao 22791 (SWFC).

**Figure 7.**
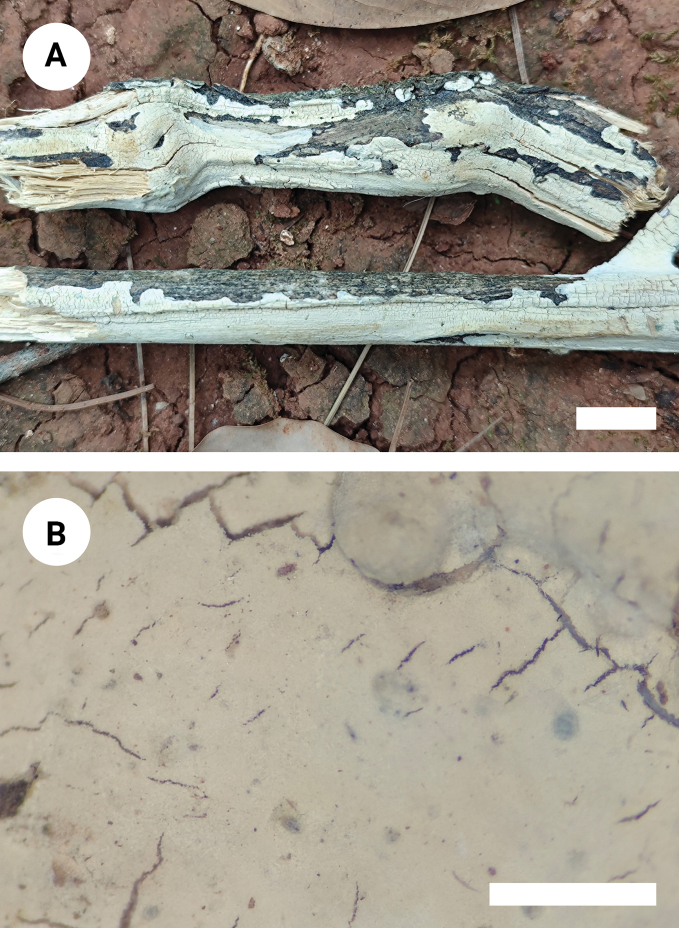
Basidiomata of *Vararialincangensis* (holotype). Scale bars: 1 cm (**A**); 1 mm **(B**).

##### Etymology.

*Lincangensis* (Lat.): referring to the locality (Lincang) of the type specimen.

**Figure 8.**
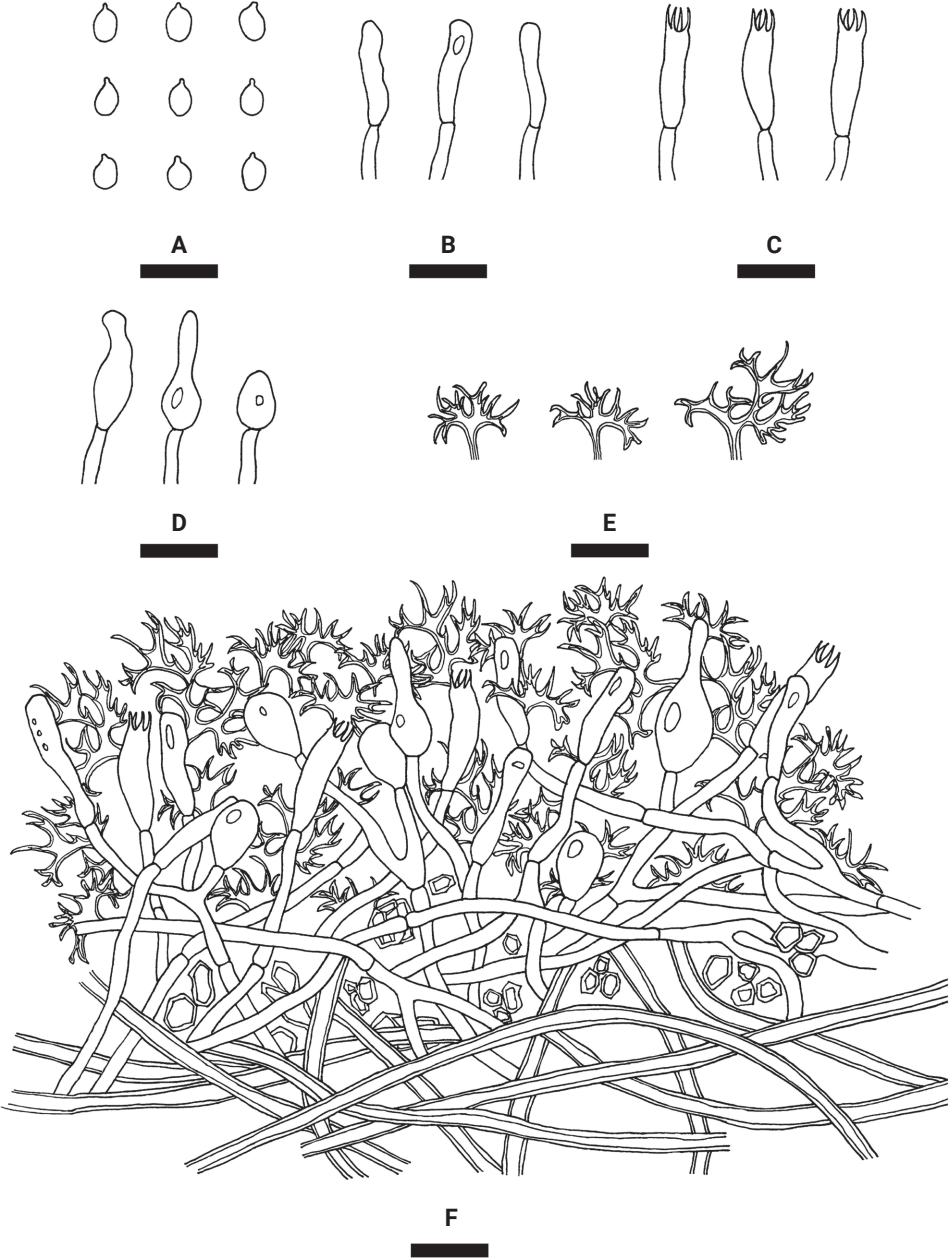
Microscopic structures of *Vararialincangensis* (holotype) **A** basidiospores **B** basidioles **C** basidia **D** gloeocystidia **E** dichohyphae **F** a section of hymenium. Scale bars: 10 µm (**A–F**).

##### Description.

Basidiomata annual, resupinate, membranous, soft and adnate, without odor or taste when fresh, up to 90 mm long, 20 mm wide, and 70–150 µm thick. Hymenial surface smooth, white to cream when fresh, cream upon drying, cracking with age. Sterile margin distinct, narrow, whitish, attached, and up to 1 mm wide.

Hyphal system dimitic, generative hyphae bearing simple-septa, rarely branched, colorless, thin-walled, 2–3 μm in diameter, IKI–, CB–, tissues unchanged in KOH; subhymenial hyphae densely covered by some crystals. Dichohyphae predominate, white to cream, capillary, thick-walled, frequently branched, dichotomously to irregularly branched with main branches and acute tips, 1–1.5 µm diameter, weakly to moderately dextrinoid in Melzer’s reagent, CB–, tissues unchanged in KOH, subiculum composed of colorless. Skeletal hyphae colorless, thick-walled, 2–3 µm in diameter, IKI–, CB–, tissues unchanged in KOH.

Gloeocystidia subglobose, and clavate to fusiform, usually containing refractive materials, colorless, smooth, thin-walled, 6.5–16 × 3–5 µm. Basidia clavate, with four sterigmata and a basal simple septum, thin-walled, smooth, 11–17.5 × 2–4 μm; basidioles in shape similar to basidia, but slightly smaller.

Basidiospores ellipsoid, colorless, thin-walled, smooth, occasionally acyanophilous, CB–, (3–)3.5–5.5(–6) × (2–)2.5–4 µm, L = 4.18 µm, W = 3.11 µm, Q = 1.33–1.36 (n = 60/2).

##### Additional specimen examined

**(paratype).** China. Yunnan Province, Lincang, Fengqing County, Yaojie Township, Xingyuan Village, 24°61'44"N, 100°17'21"E, altitude 1660 m a.s.l., on the fallen angiosperm branch, leg. C.L. Zhao, 20 July 2022, CLZhao 22799 (SWFC).

#### ﻿ Vararia punctata

Taxon classificationFungiRussulalesLachnocladiaceae

Y.L. Deng & C.L. Zhao sp. nov.

585169B0-6B32-5187-A598-39359501D2DD

MB851795

Figs 9 , 10

##### Holotype.

China. Yunnan Province, Dali, Weishan Country, Qinghua Town, Green Peacock Nature Reserve, 25°23'35"N, 100°31'39"E, altitude 1500 m a.s.l., on the fallen branch of angiosperm, leg. C.L. Zhao, 18 July 2022, CLZhao 22439 (SWFC).

**Figure 9.**
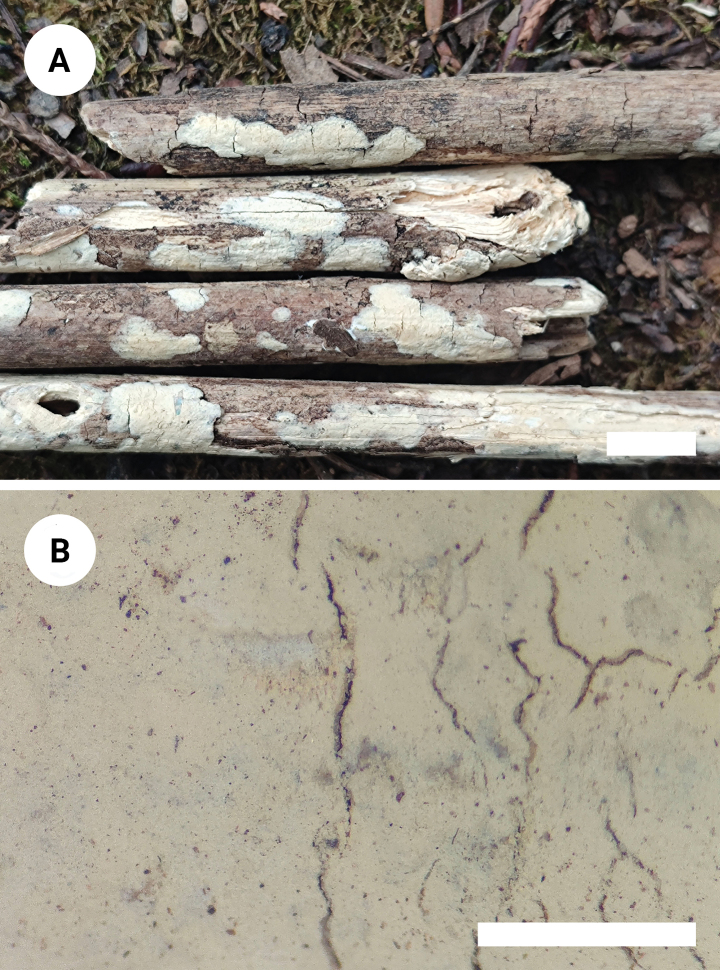
Basidiomata of *Varariapunctata* (holotype). Scale bars: 1 cm (**A**); 1 mm (**B**).

##### Etymology.

*Punctata* (Lat.): referring to the species having cushion-shaped basidioma.

**Figure 10.**
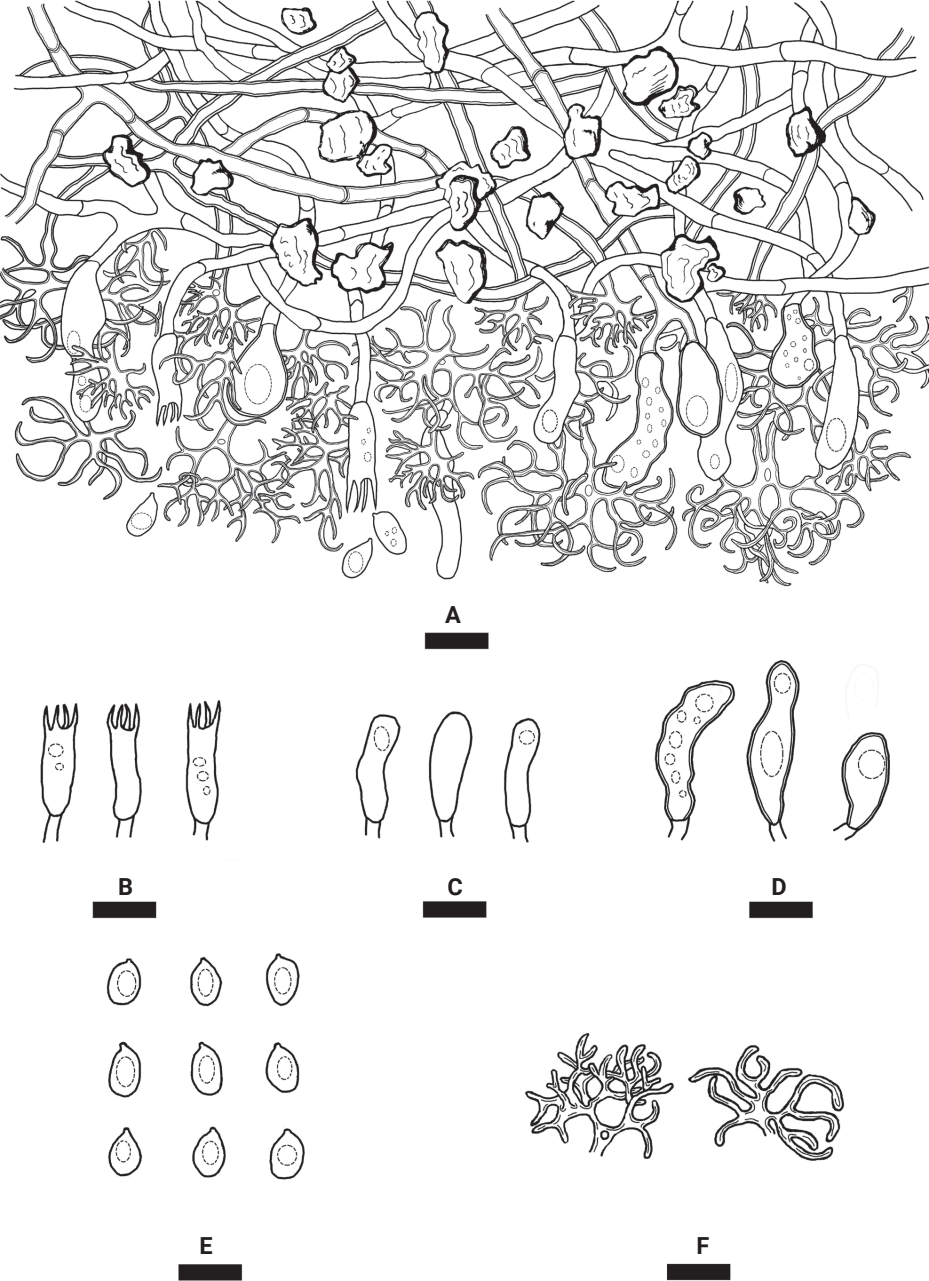
Microscopic structures of *Varariapunctata* (holotype) **A** a section of hymenium **B** basidia **C** basidioles **D** gloeocystidia **E** basidiospores **F** dichohyphae. Scale bars: 10 µm (**A–F**).

##### Description.

Basidiomata annual, membranous, soft, adnate, without odor or taste when fresh, up to 50 mm long, 15 mm wide, and 90–150 µm thick. Hymenial surface smooth, and white to cream when fresh, cream when dry. Sterile margin thin, distinct, narrow, whitish, attached, and up to 1 mm.

Hyphal system dimitic, generative hyphae bearing simple-septa, colorless, thin to slightly thick-walled, rarely branched, interwoven, 2–3 µm in diameter, IKI–, CB–, tissues unchanged in KOH. Dichohyphae predominate, white to cream, capillary, frequently branched, thick-walled, 1 µm in diameter, dichotomously to irregularly branched with main branches and acute tips, weakly to moderately dextrinoid in Melzer’s reagent, CB–, tissues unchanged in KOH. Skeletal hyphae colorless, thick-walled, 2–3 µm in diameter, IKI–, CB–, tissues unchanged in KOH; subhymenial hyphae densely covered by bulk crystals.

Gloeocystidia clavate to cylindrical, usually containing oil droplets, colorless, smooth, thick-walled, and 12–21 × 5–9 µm. Basidia subcylindrical, with four sterigmata and a basal simple septum, 11–25 × 4–7 µm; basidioles in shape similar to basidia, but slightly smaller.

Basidiospores ellipsoid, colorless, thin-walled, smooth, with oil drops, amyloid, CB–, 6–10 × 4–6(–6.5) µm, L = 7.81 µm, W = 5.1 µm, Q = 1.51–1.56 (n = 120/4).

##### Additional specimen examined

**(paratype).** China. Yunnan Province, Dali, Weishan Country, Qinghua Town, Green Peacock Nature Reserve, 25°23'35"N, 100°31'39"E, altitude 1500 m a.s.l., on the fallen branch of angiosperm, leg. C.L. Zhao, 18 July 2022, CLZhao 22423 (SWFC).

#### ﻿ Vararia sinensis

Taxon classificationFungiRussulalesLachnocladiaceae

Y.L. Deng & C.L. Zhao sp. nov.

2775F948-D649-5FA1-89BE-6924B9A89831

MB851796

Figs 11 , 12

##### Holotype.

China. Yunnan Province, Lincang, Yun County, Dumu Village, 24°39'79"N, 100°18'17"E, altitude 1960 m a.s.l., on the fallen angiosperm branch, leg. C.L. Zhao, 20 October 2022, CLZhao 25160 (SWFC).

**Figure 11.**
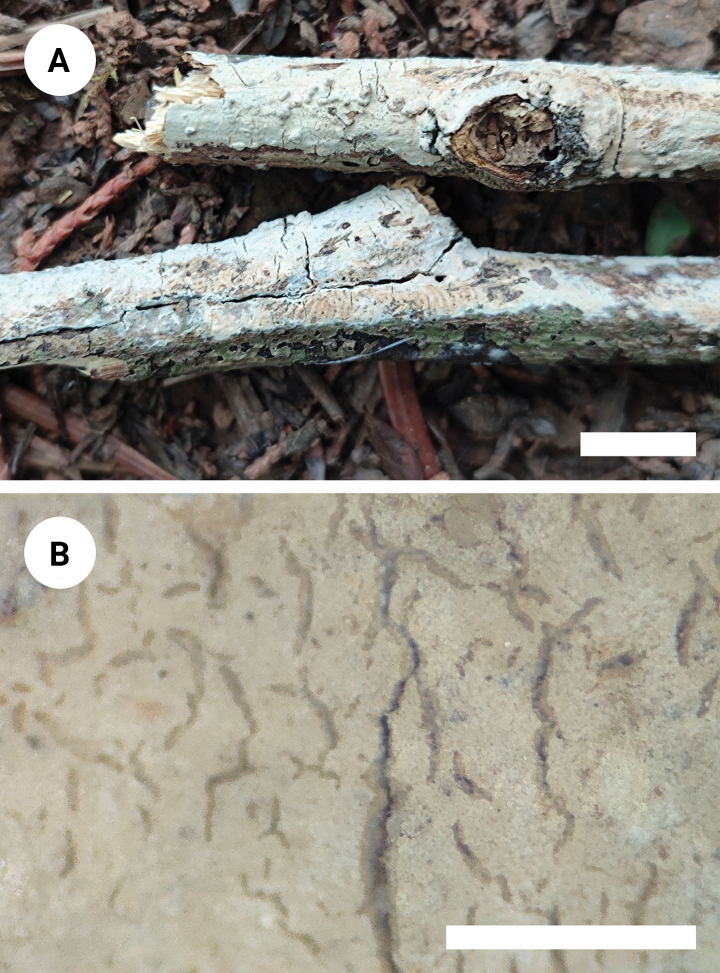
Basidiomata of *Varariasinensis* (holotype). Scale bars: 1 cm (**A**); 1 mm (**B**).

##### Etymology.

*Sinensis* (Lat.): referring to the locality (China) of the type specimen.

**Figure 12.**
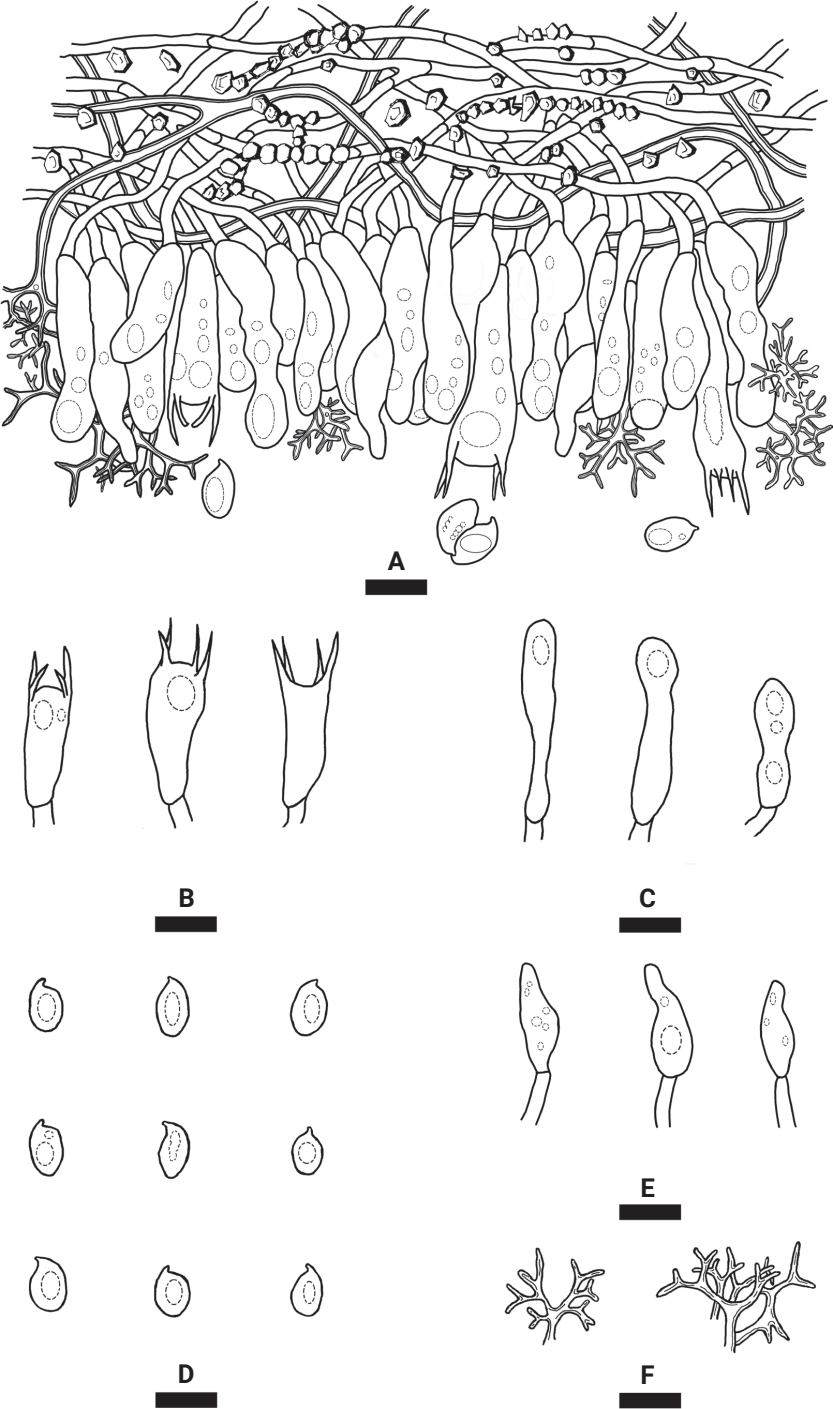
Microscopic structures of *Varariasinensis* (holotype) **A** basidiospores **B** basidioles **C** basidia **D** dichohyphae **E** gloeocystidia subulate **F** a section of hymenium. Scale bars: 10 µm (**A–F**).

##### Description.

Basidiomata annual, membranous, and adnate, up to 70 mm long, 35 mm wide, and 80–160 µm thick. Hymenial surface smooth, white to slightly pink when fresh, pink upon drying. Sterile margin thinning out, narrow, whitish, attached, and up to 1 mm.

Hyphal system dimitic, generative hyphae bearing simple-septa, colorless, thin-walled, branched, 2.5–3 µm diameter, IKI–, CB–, tissues unchanged in KOH. Dichohyphae predominant, yellowish, thick-walled, dichotomously to irregularly branched with main branches up to 1.4 μm in diameter and with acute tips, moderately dextrinoid in Melzer’s reagent, CB–, tissues unchanged in KOH, dichohyphae in hymenium similar to those in subiculum but more branched, with more narrow and shorter branches, with slightly curved tips and stronger, subhymenial hyphae densely covered by crystals. Skeletal hyphae rarely branched, interwoven, colorless, thick-walled, 2–3 µm in diameter, IKI–, CB–, tissues unchanged in KOH.

Gloeocystidia subulate, smooth, colorless, thin-walled, filled with refractive oil-like matter, 17–35 × 6–7 μm. Basidia clavate, with four sterigmata and a basal simple septum connection, 25–35 × 6–7 μm; basidioles dominant, in shape similar to basidia, but slightly smaller.

Basidiospores sub-fusiform to navicular, with a beaklike extension, colorless, smooth, with numerous oil-drops, thin-walled, IKI–, CB–, 6–11 × 4–6 µm, L = 8.21 µm, W = 4.88 µm, Q = 1.66–1.71 (n = 60/2).

##### Additional specimen examined

**(paratype).** China. Yunnan Province, Lincang, Yun County, Dumu Village. GPS coordinates: 24°39'79"N, 100°18'17"E, altitude 1960 m a.s.l., on the fallen angiosperm branch, leg. C.L. Zhao, 20 October 2022, CLZhao 25161 (SWFC).

#### ﻿ Vararia yaoshanensis

Taxon classificationFungiRussulalesLachnocladiaceae

Y.L. Deng & C.L. Zhao sp. nov.

8AC2973A-7622-5415-9A39-43841B29B00C

MB851797

Figs 13 , 14

##### Holotype.

China. Yunnan Province, Zhaotong, Qiaojia County, Yao Shan National Nature Reserve, 26°89'62"N, 102°95'04"E, altitude 2500 m a.s.l., on fallen branch of angiosperm, 21 August 2020, CLZhao 20693 (SWFC).

**Figure 13.**
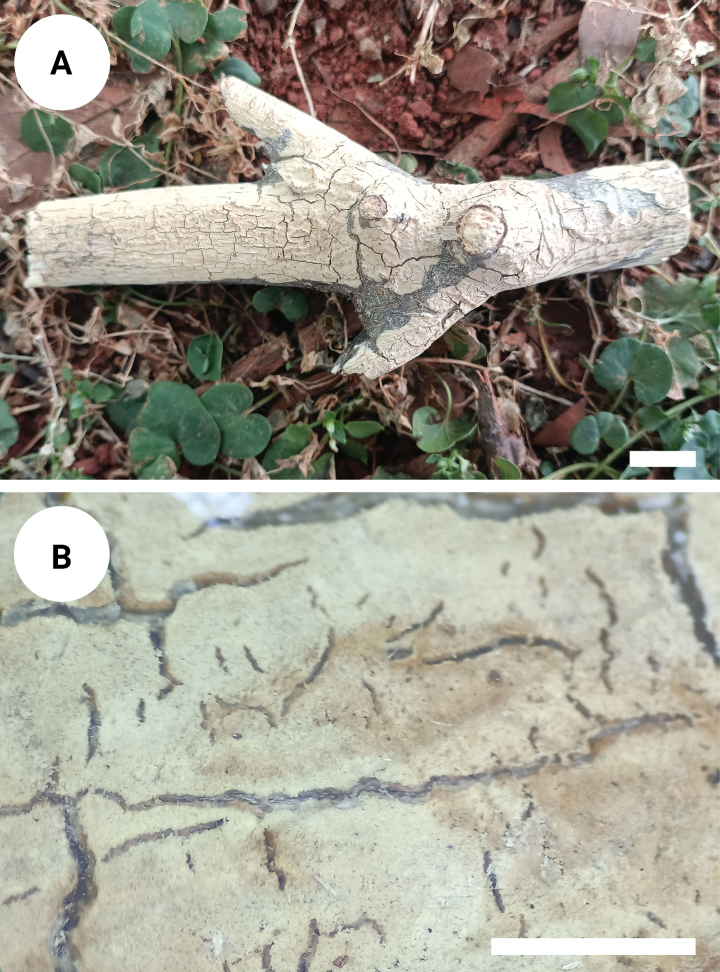
Basidiomata of *Varariayaoshanensis* (holotype). Scale bars: 1 cm (**A**); 1 mm (**B**).

##### Etymology.

*Yaoshanensis* (Lat.): referring to the provenance (Yaoshan) of the type specimen.

**Figure 14.**
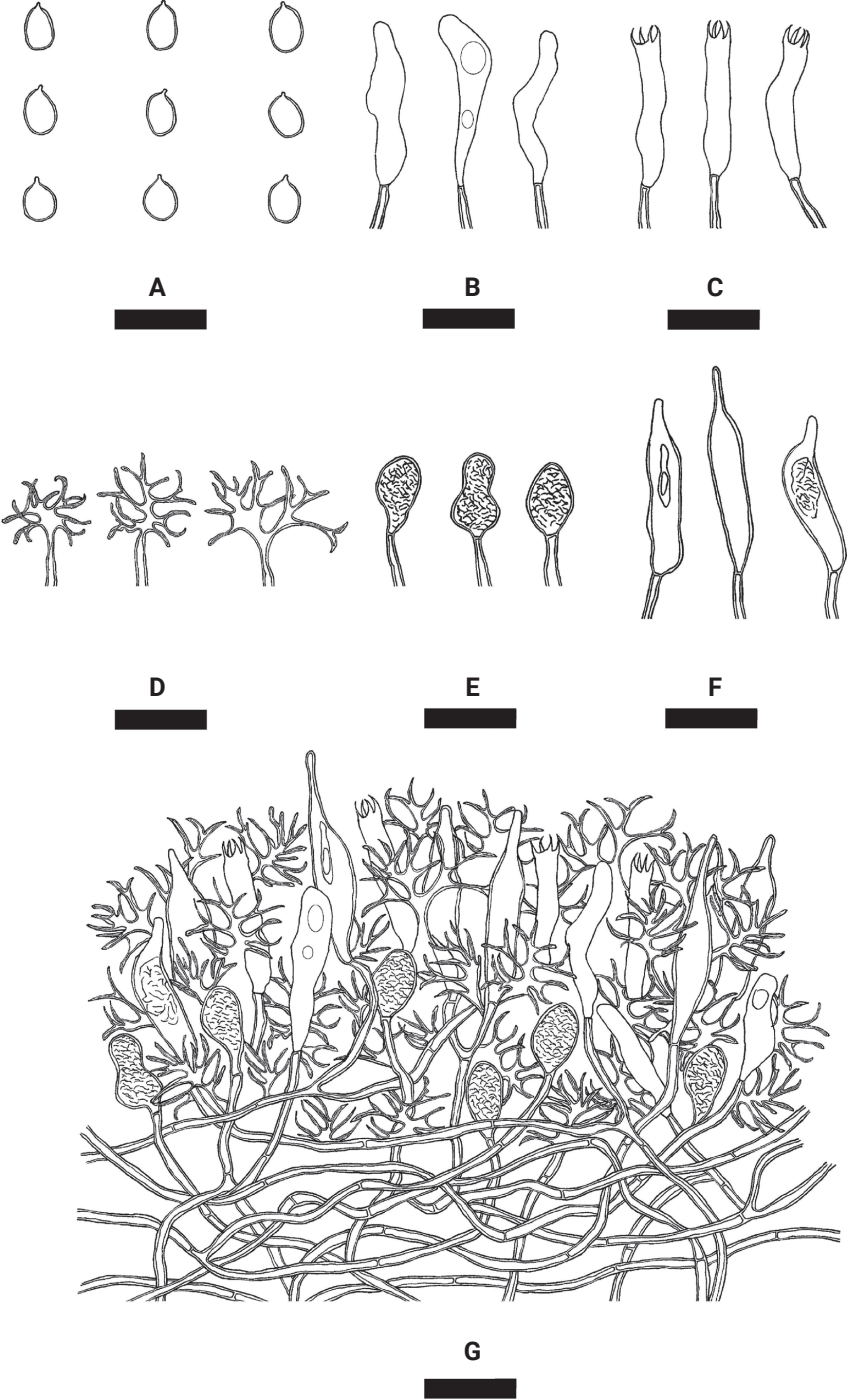
Microscopic structures of *Varariayaoshanensis* (holotype) **A** basidiospores **B** basidia **C** basidioles **D** dichohyphae **E** gloeocystidia subglobose **F** gloeocystidia clavate to fusiform **G** a section of hymenium. Scale bars: 10 µm (**A–G**).

##### Description.

Basidiomata annual, membranous, adnate, without odor or taste when fresh, up to 8 cm long, 4 cm wide, 80–120 µm thick. Hymenial surface smooth, cream to cinnamon-buff when fresh, pinkish buff to cinnamon-buff upon drying, cracking with age. Sterile margin thin, pinkish buff, up to 1 mm.

Hyphal system dimitic, generative hyphae bearing simple-septa, rarely branched, slightly thick-walled, 2–4 μm in diameter, IKI–, CB–, tissues unchanged in KOH. Dichohyphae, predominant, capillary, frequently branched, distinctly thick-walled, 1.6 µm diameter, dichotomously to irregularly branched with main branches and acute tips, weakly to moderately dextrinoid in Melzer’s reagent, CB–, tissues unchanged in KOH.

Gloeocystidia with two types, (1) Gloeocystidia fusiform, colorless, thick-walled, smooth, tapered or gradually elongated apex, 28.5–50 × 6–12.5 µm; (2) Gloeocystidia subglobose, usually containing refractive materials, colorless, thick-walled, smooth, 11–27 × 7–11 µm. Basidia are subclavate to subcylindrical, thin-walled, with four sterigmatas and a basal simple septum, 23–46 × 5–8 µm; basidioles dominant, in shape similar to basidia, but slightly smaller.

Basidiospores ellipsoid, colorless, slightly thick-walled, smooth, amyloid, CB–, (7.5–)7.6–10.8(–10.9) × (5.3–)5.7–7.8(–7.9) µm, L = 9.52 µm, W = 6.61 µm, Q = 1.4–1.5 (n = 210/7).

##### Additional specimens examined

**(paratypes).** China. Yunnan Province, Zhaotong, Qiaojia County, Yao Shan National Nature Reserve, 26°89'62"N, 102°95'04"E, altitude 2500 m a.s.l., on fallen branch of angiosperm, 21 August 2020, CLZhao 20669, CLZhao 20677, CLZhao 20697, CLZhao 20709, CLZhao 20713, CLZhao 20717 and CLZhao 20724 (SWFC), 22 August 2020, CLZhao 20528, CLZhao 20531, CLZhao 20565, CLZhao 20605, CLZhao 20608, CLZhao 20617, CLZhao 20619, CLZhao 20624, CLZhao 20646 and CLZhao 20656 (SWFC).

## ﻿Discussion

Many recently described wood-inhabiting fungal taxa have been reported worldwide, including in the genera *Vararia* (Larsson 2007; Bernicchia and Gorjón 2010; Duhem and Buyck 2012; Sanyal et al. 2012; Nakasone 2015; Liu and He 2016; Leal-Dutra et al. 2018; Liu 2019; Dai et al. 2021; Zou et al. 2022; Deng and Zhao 2023; Li et al. 2023). Prior to this study, the following eleven *Vararia* species were reported from China, *V.amphithallica* Boidin, Lanq. & Gilles, *V.bispora* S.L. Liu & S.H. He, *V.breviphysa*, *V.cinnamomea* Boidin, Lanq. & Gilles, *V.daweishanensis*, *V.fragilis*, *V.investiens*, *V.montana* S.L. Liu & S.H. He, *V.racemosa* (Burt.) D.P. Rogers & H.S. Jacks., *V.sphaericospora* Gilb. and *V.yunnanensis* Y.L. Deng & C.L. Zhao (Dai 2011; Liu 2019; Dai et al. 2021; Zou et al. 2022; Deng and Zhao 2023). The present study (Figs 1, 2) reports six new species in *Vararia*, based on a combination of morphological features and molecular evidences.

Phylogenetically, based on the multiple loci in *Scytinostroma* s.s., nine genera, *Asterostroma*, *Baltazaria*, *Dichostereum*, *Gloiothele*, *Lachnocladium*, *Michenera*, *Peniophora*, *Vesiculomyces* and *Vararia* were divided in the family Peniophoraceae (Larsson and Larsson 2003, 2004; Larsson 2007; Leal-Dutra et al. 2018; Liu and He 2018; Zou et al. 2022; Li et al. 2023). In the present study, based on the ITS+LSU data (Fig. 1), *Vararia* was grouped with *Asterostroma*, *Baltazaria*, *Dichostereum*, and *Peniophora*, in which six new species were grouped into the genus *Vararia*. From the phylogram inferred from the ITS+LSU data (Fig. 1), the four new species *V.fissurata*, *V.punctata*, *V.isabellina* and *V.sinensis* were retrieved as a sister to *V.ellipsospora*, *V.ambigua*, *V.investiens* and *V.breviphysa*, respectively. Furthermore, the two new species *Vararialincangensis* and *V.yaoshanensis* formed a monophyletic lineage respectively, and then *V.yaoshanensis* was clustered with *V.ellipsospora* and *V.tropica*. The species *V.lincangensis* was grouped closely with *V.ambigua*, *V.gallica* and *V.punctata*. However, morphologically, *V.investiens* can be delimited from *V.isabellina* by having the resupinate basidiomata with the yellowish cream to ochraceous hymenial surface, thin-walled, clamped generative hyphae, longer fusiform gloeocystidia (40–80 × 4–8 µm), longer basidia (30–50 × 4–5 µm), and smaller fusoid basidiospores measuring as 8–12 × 3–3.5 µm (Boidin and Lanquetin 1975). The taxon *V.ellipsospora* is different from *V.yaoshanensis* by having the smaller cylindrical basidia (24–30 × 5–6 µm), longer gloeocystidia (40–56 × 8–10 µm), and narrower basidiospores (8–12 × 5.5–6.5 µm; Cunningham 1955), and *V.tropica* is distinguished from *V.yaoshanensis* by its smaller subcylindrical gloeocystides (20–42 × 6.5–10 µm), and larger basidia (35–50 × 7–8.5 µm; Welden 1965). In addition, *V.ambigua* differs from *V.lincangensis* by having both larger gloeocystidia (15–32 × 3.5–7 µm), and basidiospores measuring as 6–7.3 × 3.4–5 µm (Boidin et al. 1980); *V.gallica* is different from *V.lincangensis* by its larger fusiform gloeocystidia (15–36 × 3.5–6.5 µm) and basidiospores (9–12 × 3.5–5 µm; Boidin and Lanquetin 1975; Grosse-Brauckmann and Kummer 2004).

Based on ITS topology (Fig. 2), the present study highlighted that *V.fissurata* was found to be the sister to *V.ellipsospora* with strong supports, and morphologically *V.ellipsospora* is different from *V.fissurata* by the fimbriate basidiomata, thick-walled generative hyphae, larger flexuous-cylindrical gloeocystidia (40–56 × 8–10 µm), longer basidia (24–30 × 5–6 µm), and longer oblong ellipsoid basidiospores (8–12 × 5.5–6.5 µm; Cunningham 1955). In addition, *V.lincangensis* was clustered with *V.fragilis*, but morphologically *V.fragilis* is distinguished from *V.lincangensis* by the brittle basidiomata, with a buff to ochraceous hymenial surface and elliptical to ovoid gloeocystidia, both larger subulate gloeocystidia (16.5–27 × 4–7 µm) and subcylindrical basidia (13–23.5 × 3–4.5 µm; Zou et al. 2022). Furthermore, *V.punctata* was retrieved as a sister to *V.ambigua*, but morphologically *V.ambigua* differs from *V.punctata* by its cream to buff hymenophore, and larger fusiform gloeocystidia measuring as 15–32 × 3.5–7 µm (Boidin et al. 1980). Further, *V.isabellina* formed a monophyletic lineage and then was grouped closely with *V.daweishanensis* and *V.gracilispora* Boidin & Lanq. However, morphologically *V.daweishanensis* is distinguishable from *V.isabellina* by its pale yellowish hymenial surface, clamped generative hyphae, and smaller gloeocystidia (9–23 × 7–10.5 µm), longer basidia measuring as 26–46 × 5–8 µm, narrower allantoid basidiospores (9–13 × 3.5–5 µm; Zou et al. 2022). Moreover, *V.sinensis* was grouped with five taxa: *V.breviphysa*, *V.pirispora*, *V.fusispora*, *V.abortiphysa*, and *V.insolita*, however, morphologically, *V.breviphysa* is distinguishable from *V.sinensis* by having light yellow to light brown basidiomata, larger subcylindrical gloeocystides (50–65 × 6–8.5 µm), larger basidia (30–38 × 5.5–7 µm), and longer fusiform basidiospores (16–20 × 4–5 µm, Boidin and Lanquetin 1975; Liu et al. 2019); the species *V.pirispora* is distinct from *V.sinensis* by its larger subcylindrical gloeocystides (40–65 × 6–8 µm), longer basidia measuring as 36–52 × 6–7 µm, larger pyriform basidiospores (10–16.5 × 5–7 µm; Boidin et al. 1987); *V.fusispora* can be delimited from *V.sinensis* by having larger cylindrical gloeocystidia (40–60 × 5–6 µm) and oval to fusiform gloeocystidia (24–60 × 6–12 µm), subclavate basidia (35–56 × 6–9 µm), and larger fusiform basidiospores measuring as 14–17 × 4–6 µm (Cunningham 1955); *V.abortiphysa* is distinct from *V.sinensis* by its plagio and subcylindrical gloeocystides measuring as 25–45 × 4.5–9 µm, and longer cylindrical basidiospores (14–17 × 2.2–2.8 µm; Boidin and Lanquetin 1975); *V.insolita* is distinguishable from *V.sinensis* by having larger gloeocystidia measuring as 60–80 × 5–8 µm, longer subcylindrical basidia (30–78 × 5.5–6.5 µm), and longer subfusiform basidiospores (12–16 × 4.2–5.75 µm; Boidin and Lanquetin 1975). Then *V.yaoshanensis* was found to be the sister to *V.gallica* (Bourdot & Galzin) Boidin with strong supports. However, morphologically, *V.gallica* can be delimited from *V.yaoshanensis* by its thin-walled generative hyphae, smaller thin-walled fusiform gloeocystidia (15–36 × 3.5–6.5 µm), and thin-walled, narrower basidiospores measuring as 9–12 × 3.5–5 µm (Boidin and Lanquetin 1975; Grosse-Brauckmann and Kummer 2004).

Based on our phylogenetic and morphological research results, 17 species have been reported from China, including newly described in the present study and other recently published papers in this country (Dai 2011; Liu and He 2016; Liu 2019; Dai et al. 2021; Zou et al. 2022; Deng and Zhao 2023). It seems that the species diversity of *Vararia* is rich in China. Although *Vararia* taxa are well studied in the present paper, the species diversity, taxonomy and phylogeny of *Vararia* and related genera are still unresolved. A comprehensive study on this issue is urgently needed.

### ﻿A key to 17 species of *Vararia* s.l. in China

**Table:** 

1	Generative hyphae with clamp connections	**2**
–	Generative hyphae bearing simple-septa	**3**
2	Basidia with 2 sterigmatas	**4**
–	Basidia with 4 sterigmatas	**5**
3	Present thick-walled skeletal hyphae	**6**
–	Absent thick-walled skeletal hyphae	**7**
4	Subcylindrical to fusiform basidiospores measuring as (10.5–)12–17(–20) × 4.5–5.5(–6.5) µm, slightly thick-walled, subglobose gloeocystidia (15–30(–35) × 6–8(–10) µm), and subcylindrical or gradually narrower gloeocystidia (25–40(–65) × 4.5–6(–18) µm)	***V.amphithallica***
–	Fusiform to cylindrical basidiospores measuring as (16–)18–22(–14) × 6–7.2(–8) µm, thick-walled, ventricose, gloeocystidia with an apical papilla (20–40 × 9–12 µm)	***V.bispora***
5	Thin to thick-walled generative hyphae, subcylindrical basidia (26–46 × 5–8 µm), allantoid basidiospores measuring as (8.5–) 9–13 (–14) × 3.5–5 µm, and ellipsoid to ovoid to subcylindrical gloeocystidia (9–23 × 7–10.5 µm)	***V.daweishanensis***
–	Thin-walled generative hyphae	**8**
6	Thin to slightly thick-walled generative hyphae, thick-walled, clavate to cylindrical gloeocystidia (12–21 × 5–9 µm), subcylindrical basidia (11–25 × 4–7 µm), and ellipsoid basidiospores (6–10 × 4–6(–6.5) µm)	***V.punctata***
–	Thin-walled generative hyphae, clavate basidia	**9**
7	Slightly thick-walled generative hyphae	**10**
–	Thin-walled generative hyphae	**11**
8	Gloeocystidia two kinds	***V.fissurata***
–	Gloeocystidia one kinds	**12**
9	Ellipsoid basidiospores measuring as (3–)3.5–5.5(–6) × (2–)2.5–4 µm, subglobose, clavate to fusiform gloeocystidia (6.5–16 × 3–5 µm)	***V.lincangensis***
–	Subfusiform to navicular basidiospores (6–11 × 4–6 µm), subulate gloeocystidia (17–35 × 6–7 µm)	***V.sinensis***
10	Slightly thick-walled, ellipsoid basidiospores measuring as (7.5–)7.6–10.8(–10.9) × (5.3–)5.7–7.8(–7.9) µm, thick-walled, fusiform gloeocystidia (28.5–50 × 6–12.5 µm), globose gloeocystidia (11–27 × 7–11 µm), subclavate to subcylindrical basidia (23–46 × 5–8 µm)	***V.yaoshanensis***
–	Thin-walled basidiospores, subcylindrical basidia	**13**
11	Slightly thick-walled, ellipsoid basidiospores measuring as (5.1–)5.9–11.5(–11.8) × (4.3–)4.7–8.6(–9) µm, cylindrical basidia (17.5–32 × 5–9.5 µm), thin- to slightly thick-walled, subcylindrical gloeocystidia (16.5–58.5 × 4–10 µm), fusiform gloeocystidia (18.5–43.5 × 7–9 µm), tapering gloeocystidia (27.5–42 × 5.5–9 µm)	***V.yunnanensis***
–	Thin-walled basidiospores	**14**
12	Basidiospores < 5 µm in diameter	***V.investiens***
–	Basidiospores > 5 µm in diameter	**15**
13	Broad ellipsoid to ellipsoid basidiospores measuring as 3.5–5.5(–6) × 2.5–3.5 µm, elliptical to ovoid gloeocystidia (5.8–16 × 3.5–7 µm), subulate gloeocystidia (16.5–27 × 4–7 µm)	***V.fragilis***
–	Sub-fusiform to navicular basidiospores with numerous oil-drops measuring as 9–13 × 5–8 µm, spindle to subcylindrical gloeocystidia (38–47 × 8–13 µm)	***V.isabellina***
14	Rose to orange subfusiform basidiospores measuring as (14–)16–19(–21.5) × 4.2–6 µm, cylindrical basidia (30–53 × 6.5–7.5 µm), thick-walled, subcylindrical Gloeocystides (50–65 × 6–7(–8.5) µm)	***V.breviphysa***
–	Colorless basidiospores	**16**
15	Broadly ellipsoid basidiospores measuring as (11–)12–16(–17) × (7.5–)9.5–13(–14) µm, clavate basidia (70–110 × 10–16 µm), clavate gloeocystidia (50–100 × 4–9 µm)	***V.montana***
–	Spherical basidiospores measuring as 8–10 × 7.5–8.5 µm, cylindrical to clavate basidia (40–45 × 6–7.5 µm), subcylindrical to fusiform gloeocystides (48–80(–105) × 7–11(–14) µm)	***V.sphaericospora***
16	Subcylindrical to fusiform gloeocystides (26–40 × 4.5–9 µm), cylindrical basidiospores (6–8 × 2–3 µm), cylindrical basidia (30–40 × 4–5 µm)	***V.racemosa***
–	Absent gloeocystides, oblong to subellipsoid basidiospores measuring as 9–13 × 5–7.2 µm, and subcylindrical basidia (45–65 × 8–10 µm)	***V.cinnamomea***

## Supplementary Material

XML Treatment for Vararia fissurata

XML Treatment for Vararia isabellina

XML Treatment for Vararia lincangensis

XML Treatment for Vararia punctata

XML Treatment for Vararia sinensis

XML Treatment for Vararia yaoshanensis

## ﻿Additional information

### Author ORCIDs

Yinglian Deng https://orcid.org/0000-0002-8220-508X

Sana Jabeen https://orcid.org/0000-0001-8839-7716

Changlin Zhao https://orcid.org/0000-0002-8668-1075

### Data availability

All of the data that support the findings of this study are available in the main text.
